# Supplementary material for: Anisotropic long-range spin transport in canted antiferromagnetic orthoferrite YFeO3
Source: Nat Commun. 2022 Oct 17;13:6140. doi: 10.1038/s41467-022-33520-5 (PMC9576681; doi:10.1038/s41467-022-33520-5)
Supplement: Supplementary file 1 — Supplementary Information [file 41467_2022_33520_MOESM1_ESM.pdf]

Supplementary - Anisotropic long-range spin transport in canted antiferromagnetic orthoferrite $YFeO_3$

Shubhankar Das[1], A. Ross[2,$], X. X. Ma[3,$], S. Becker[1], C. Schmitt[1], F. van Duijn[4,5], E. F. Galindez-Ruales[1], F. Fuhrmann[1], M.-A. Syskaki[1], U. Ebels[4], V. Baltz[4], A.-L. Barra[5], H. Y. Chen[3], G. Jakob[1,6], S. X. Cao[3*], J. Sinova[1], O. Gomonay[1], R. Lebrun[2], and M. Kläui[1,6,7*]

[1]Institute of Physics, Johannes Gutenberg University Mainz, Staudingerweg 7, 55128 Mainz, Germany

[2]Unité Mixte de Physique CNRS, Thales, Université Paris-Saclay, Palaiseau 91767, France

[3]Department of Physics, Materials Genome Institute, International Center for Quantum and Molecular Structures, Shanghai University, Shanghai, 200444, China

[4]Univ. Grenoble Alpes, CNRS, CEA, Grenoble INP, SPINTEC, F-38000 Grenoble, France

[5]Laboratoire National des Champs Magnétiques Intenses, CNRS-UGA-UPS-INSA-EMFL, F-38042 Grenoble, France

[6]Graduate School of Excellence Materials Science in Mainz, Staudingerweg 9, 55128 Mainz, Germany

[7]Center for Quantum Spintronics, Norwegian University of Science and Technology, Trondheim 7491, Norway

*Corresponding authors – M. Kläui (klaeui@uni-mainz.de) and S. X. Cao (sxcao@shu.edu.cn)

[$]these authors contributed equally

Single crystal sample preparation. Y_2O_3 (4N) and Fe_2O_3 (3N) powders were used as raw materials to make the polycrystalline sample according to the stoichiometric ratio by the conventional solid-state reaction method. The original reagents were weighed carefully and pulverized with moderate anhydrous ethanol in an agate mortar. The mixtures were sintered at 1250 °C for 1000 minutes to guarantee an adequate reaction and then furnace-cooled to room temperature. To ensure sufficient reaction, we continue to grind the pre-sintered sample into powders, and then it is pressed into a thin sheet of about 1.5 mm thickness to narrow the gap between the powder particles for secondary sintering. The sintering temperature and duration are the same as for the pre-fired process. The secondary sintered samples were thoroughly reground, and the polycrystalline powders were pressed into two rods that are 35-85 mm in length and 5-6 mm in diameter by Hydrostatic Press System at 60 MPa, and then sintered again at 1250 °C for sufficient reaction.

High-quality $YFeO_3$ single crystals were then successfully grown by an optical floating zone furnace (Crystal System Crop, model FZ-T-10000-H-VI-P-SH) from the sintered polycrystalline sample. In the process of crystal growth, both the upper and lower rods rotate at the opposite direction of 15 rpm at the same time, and the molten zone moves upward at a speed of 2 mm/h. The picture of the $YFeO_3$ single crystal is shown in Supplementary Figure 1.

The crystallographic orientations of the $YFeO_3$ single crystal were determined by back-reflection Laue X-ray photography. Clear and sharp Laue spots imply a high quality of the single crystal. The back-reflection Laue images of the $YFeO_3$ single crystal along the *a*-axis, *b*-axis, and *c*-axis are shown in Supplementary Figure 2. Then samples of $YFeO_3$ single crystal perpendicular to the different symmetry axes are cut from the single crystal, whose accurate direction is further confirmed by four-cycle X-ray diffraction (XRD) measurement.

Structural characterisation. The crystallographic properties were investigated using XRD. The sample has been fixed to an aluminium plate and measured in a Bruker D8 equipped with a copper anode, Göbel mirror and a monochromator. The sample has first been aligned with respect to the sample surface in the setup. The (010) reflex has then been found by tilting the sample by 11° out of the initial position. This indicates that the sample has a miscut. As a result, the c- and a-axes are also misaligned with the surface by around 8° as shown in Supplementary Figure 3(a). The crystallographic purity has been confirmed by performing 2θ/ω measurements along the (010), (110) and (021) direction. An example scan along the (010) direction is shown in Supplementary Figure 3(b). No other peaks except the expected $YFeO_3$ peaks are found in these scans, indicating the absence of foreign phases or twinned crystallographic domains. The calculated lattice parameters of a = 5.28 Å, b = 5.57 Å and c = 7.6 Å are in line with other reports[1].

Magnetic Characterization. To check the magnetic properties of the $YFeO_3$ crystal, *m-H* hysteresis curves have been measured at 300 K by applying the field along the c-axis (see Supplementary Figure 4). We observe a saturation magnetization of around 0.05 μ_B/f.u., which is relatively large compared to that of bulk α-Fe_2O_3, that can be understood from the large Dzyaloshinskii–Moriya interaction field of 12 T leading to a canting of the antiferromagnetically aligned sublattices. A sharp transition between the magnetization direction is observed with a coercive field of 45 ± 5 mT.

Antiferromagnetic resonance. Supplementary Figures 6 and 7 show the resonance peak at different frequencies of the low frequency magnon eigenmode for fields applied along the c-axis at 20 K and 150 K, respectively. Supplementary Figures 8 and 9 show the same measurements but the field is applied along a-axis at 20 K and 150 K, respectively. The multiple resonance peaks appear because the wavelength is far smaller than the thickness of the crystal.

The frequency dependence is fitted using the model described in the main text and Ref. 2. Supplementary Figure 5 shows linewidth as a function frequency for field applied along a-axis at 20 K.

Thermal spin transport for field applied along the easy-axis. The flowing charge current in the Pt-injector leads to Joule heating, creating a lateral temperature gradient between the Pt wires. This gradient results in a thermal spin current induced by the spin Seebeck effect[3,4]. The thermal magnon spin current flowing in the $YFeO_3$ is then absorbed by a spatially and electrically separated Pt detector, where it is converted to a measurable voltage V_{th} by the inverse-SHE[5,6]. As V_{th} does not depend on current polarity in the injector, we calculate the V_{th} signal by adding the voltage at the detector due to the opposite polarity current in injector[6,7]; $V_{th} = (V(I+) + V(I-))/2$. We then express the non-local signal due to thermal transport as $R_{th} = V_{th}/I^2$ (in units of VA^{-2}).

Supplementary Figure 10(a) shows thermal spin signal R_{th} as a function of field for device where wires are along the easy-axis. On applying the magnetic field, R_{th} increases and reaches maximum at a field strength below H_{cr}, followed by a sharp decrease on further increasing the field. The peak and the decrease in R_{th} replicate the electrical transport signal R_{el} (see Figure 2(a) of main text) and can be explained from the equilibrium orientation of Néel vector (**n**) as described in the main text. This reveals that the dominant contribution of thermal spin transport is also mediated by Néel vector. Both the non-zero R_{th} signal and the linear increase with field above H_{cr} are not directly related to the spin orientation but could have origins in emerging field induced magnetic moments, Hanle effect, thermal Hall effect, Seebeck effect and interfacial spin Seebeck effect at the detector etc.[8-11]. The necessary data to be able to disentangle every single effect goes beyond the scope of this work and could be a unique study alone.

Supplementary Figure 10(b) shows field dependence of R_{th} for device where wires are perpendicular to the easy-axis. The R_{th} shows distinct behaviour; starting with zero R_{th} signal in absence of field, it increases significantly with field followed by a maximum at $\mu_0 H = 3$ T, then turn to decrease and shows a change of sign. Finally, at $H > H_{cr}$ the gradient of the signal reverses and signal amplitude linearly increases with field. The change in sign of R_{th} at 6 T and the linear increase above H_{cr} are the outcome of the emergent dominant contribution from various mechanisms mentioned in previous geometry (shown in Supplementary Figure 10(a)). The contribution to R_{th} from those various mechanisms is opposite in sign to the contribution coming from Néel vector in this geometry[8-11]. The solid lines in Supplementary Figures 10(a) and (b) indicate the transport expectation from our model, showing excellent agreement.

Spin transport for field perpendicular to the easy-axis. Supplementary Figures 11 (a) and (b) show the R_{el} and R_{th} signal as a function of field applied perpendicular to the easy-axis for the device where wires are parallel to the easy-axis. No R_{el} signal is observed in the whole field range, whereas the gradual increase of R_{th} and saturation tendency at high field indicates that the field induced net moment contributes to thermal spin transport[8-10].

Supplementary Figures 12 (a) and (b) show R_{el} and R_{th} signals as a function of field applied perpendicular to the easy-axis for the device where wires are perpendicular to the easy-axis. On applying the field perpendicular to the easy-axis, we didn't observe any R_{el} signal due to the non-rotation of **n** which prefers to stay perpendicular to the field. This indicates that the field perpendicular to **n** is unable to create the ellipticity in the magnon modes that is required to transport spin angular momentum (see discussion section in the main text and below for details). A very weak linear field dependence of R_{th} is observed which may arise from the misalignment of crystallographic axes with the sample plane due to crystal miscut (see Supplementary Figure 3(a)).

Angular dependence of spin transport for wires perpendicular to the easy-axis. Supplementary Figures 13(a) and (b) show the angular dependence of the R_{el} and R_{th} signal, respectively, for various fields, where α is defined as the angle between the current direction (c-axis) and the field. The R_{th} vs α curve shows the expected cos(α) dependence at 3 T, but the angular dependence below and above the critical field show a reverse gradient. The solid lines are the fitting based on magnon dynamics considered in the proposed theoretical model.

Temperature dependence of the spin transport. We have studied the field dependence of spin transport signal for various temperatures for device where wires perpendicular to the easy-axis. Supplementary Figure 14 shows the R_{el} signal as a function of temperature at $\mu_0 H = 3$ T (the field where maximum of R_{el} vs. H curve is observed). With increasing the temperature, R_{el} signal increases and shows a maximum value around 200 K and further decreases with higher temperature. The peak around 200 K can be explained from the temperature dependence competition between magnon decay length and magnon population. With decreasing temperature, the magnon decay length increases[2] whereas the magnon population decreases. We could not detect any signal below 50 K within our measurement accuracy. The absence of detectable signal at low temperature indicates the diffusive nature of the transport and excludes spin-superfluidity as the dominant mode of transport.

Theoretical Model

For the description of the magnetic dynamics, we use the same approach as suggested in Ref. 2. We use the standard dynamical equation for the Néel vector (**n**, $|\mathbf{n}| = 1$), which is valid on the assumption of small spin canting

$$\mathbf{n}\times\left[\ddot{\mathbf{n}} - 2\gamma\dot{\mathbf{n}}\times\mathbf{H} + \gamma\alpha_G H_{ex}\dot{\mathbf{n}} - \nabla\cdot\hat{c}^2\nabla\mathbf{n} + \gamma^2\frac{H_{ex}}{M_s}\frac{\partial w_{AF}}{\partial\mathbf{n}}\right] = \gamma^2 H_{ex}\mathbf{n}\times\mathbf{H}_{curr}\times\mathbf{n}. \quad (1)$$

Here $\hat{c}$ = c_a, c_b, c_c is the diagonal tensor of the limiting magnon velocity along different crystallographic axes, γ is the gyromagnetic ratio, α_G is the Gilbert damping coefficient, H_{ex} is the exchange field that keeps the magnetic sublattice moments antiparallel, $\mathbf{H}_{curr} = \hbar\varepsilon\theta_H \mathbf{j}\times\hat{z}/(2ed_{AF}M_s)$ is directed along the spin accumulation direction $\hat{\mu}$ ($|\hat{\mu}| = 1$) of the current in the Pt electrode, $\mathbf{j}$ is the current density, $\hbar$ is the Planck constant, d_{AF} is the penetration depth of the spin current into the orthoferrite, $0 < \varepsilon \leq 1$ is the spin-polarization efficiency, θ_H is the spin Hall angle, e is the electron charge, and $M_s/2$ is sublattice magnetization. The expression for the magnetic energy density w_{AF} ($\mathbf{n}$; $\mathbf{H}$) in the presence of the constant external magnetic field $\mathbf{H}$ can be written as[12,13]

$$w_{\mathrm{AF}}(\mathbf{n}) = -\frac{1}{2}M_s H_a n_x^2 + \frac{1}{2}M_s H_b n_z^2 + \frac{M_s}{2H_{ex}}(\mathbf{H}\cdot\mathbf{n})^2 + \frac{M_s}{H_{ex}}H_{DMI}\hat{z}\cdot\mathbf{n}\times\mathbf{H}\,, \tag{2}$$

where $H_a > 0$ and $H_b > 0$ are anisotropy fields associated with the easy (a-axis) and hard (b-axis) magnetic direction. $H_{DMI} > 0$ is the homogeneous DMI field responsible for a small spin canting (and finite magnetization) along the c-axis. Orthogonal coordinates are associated with the crystallographic axes: $\hat{x} \parallel \mathbf{a}$, $\hat{y} \parallel \mathbf{c}$, $\hat{z} \parallel \mathbf{b}$.

For modelling we use the following values of the parameters: $\mu_0 H_{DMI} = 12$ T, $\mu_0 H_a = 0.19$ T, $\mu_0 H_{cr} = 6.5$ T, and $\mu_0 H_{ex} = 635$ T, consistent with the values extracted from AFMR measurements. The anisotropy field along the b-axis was estimated as $\mu_0 H_b = 0.7$ T, to satisfy the ratio between low and high frequencies magnon eigenmodes observed in Ref. [14,15].

Equilibrium state. The equilibrium orientation of the Néel vector, $\mathbf{n}^{(0)}(\mathbf{H})$, in the presence of the field applied parallel to the easy-axis, $\mathbf{H} \parallel \hat{x}$, rotates smoothly within the xy plane[12], so that

$$n_y^{(0)}(H_x) = \frac{H_{DMI}H_x}{H_a H_{ex} - H_x^2}\,. \tag{3}$$

At critical field

$$H_{cr} = -\frac{1}{2}H_{DMI} + \sqrt{H_a H_{ex} + \frac{1}{4}H_{DMI}^2} \;, \tag{4}$$

the Néel vector reaches perpendicular to the magnetic field and parallel to the intermediate anisotropy axis $c \parallel \hat{y}$. Further increasing the magnetic field ($H_x > H_{cr}$) produces only a canting of magnetic sublattices resulting in a nonzero magnetization,

$$m_x = M_s \frac{H_{DMI} + H_x}{H_{ex}}. \tag{5}$$

This behaviour of the Néel vector contrasts with the abrupt spin-flop transition, which is observed in compensated antiferromagnets, and is governed by DMI.

Spin transport. To study the magnon spin transport, we start from calculations of magnon spectra in presence of the external magnetic field. For this we consider small fluctuations $\delta\mathbf{n}$ of the Néel vector on top of an equilibrium state $\mathbf{n}^{(0)}$: $\mathbf{n} = \mathbf{n}^{(0)}(\mathbf{H}) + \delta\mathbf{n}$, $\delta\mathbf{n} \perp \mathbf{n}^{(0)}$ and assume that $\delta\mathbf{n}(t, \mathbf{k}) \propto \exp(-i\omega t + i\mathbf{k}\cdot\mathbf{r})$. The magnons are then calculated as eigenmodes of the linearized equation (1) that takes the form

$$\begin{aligned} &\delta\ddot{n}_1 - 2\omega_H \delta\dot{n}_2 - \sum_{j=1}^{3} c_j^2 \frac{\partial^2}{\partial x_j^2}\delta n_1 + \omega_1^2 \delta n_1 = 0 \\ &\delta\ddot{n}_2 + 2\omega_H \delta\dot{n}_1 - \sum_{j=1}^{3} c_j^2 \frac{\partial^2}{\partial x_j^2}\delta n_2 + \omega_2^2 \delta n_2 = 0, \end{aligned} \tag{6}$$

where $\delta n_{1,2}$ and $\omega_{1,2}^2$ are the eigen-vectors and eigen-values of the matrix $\gamma^2 H_{ex} M_s \left(\partial^2 w_{AF} / \partial n_j \partial n_k\right)|_{\mathbf{n}^{(0)}}$, $\omega_H = \gamma \mathbf{H}\cdot\mathbf{n}^{(0)}$. The eigen-frequencies of two magnons eigenmodes with $\mathbf{k} = 0$ are given by the expression

$$\omega_{\pm}^2 = \frac{1}{2}\left(\omega_1^2 + \omega_2^2\right) + 2\omega_H^2 \pm \sqrt{\frac{1}{4}\left(\omega_1^2 - \omega_2^2\right)^2 + 2\omega_H^2\left(\omega_1^2 + \omega_2^2\right) + 4\omega_H^4} \,. \tag{7}$$

Frequencies of the modes with nonzero $\mathbf{k}$ are obtained by substitution $\omega_{1,2}^2 \rightarrow \omega_{1,2}^2 + \sum_{j=1}^{3} c_j^2\, k_j^2$. This approach is appropriate for magnons with $\mathbf{k}$ vectors far from the Brillouin zone

edge which give the main contribution to the observed spin transport. Figure 4(a) of the main text shows the field dependence of eigen-frequencies $\omega_{\pm}(H)$ calculated for field along the easy-axis consistent with the previous reports[14]. The low frequency mode vanishes at the critical field $H = H_{cr}$, and the high frequency mode has nonzero frequency which slightly decreases above H_{cr}.

Next, we discuss the ability of eigen modes to transport spin. The angular momentum of an eigenmode is a vector parallel to the dynamical magnetization[16]

$$\mathbf{m}_{dyn} = M_s \frac{\delta\mathbf{n} \times \delta\dot{\mathbf{n}}}{\gamma H_{ex}}. \quad (8)$$

From the orthogonality condition $\delta\mathbf{n} \perp \mathbf{n}^{(0)}$, it follows that $\mathbf{m}_{dyn} \parallel \mathbf{n}^{(0)}$. We consider only the modes with stationary magnetization, as only these modes contribute to spin transport signal.

As follows from equation (8), magnon magnetization depends on the polarization of the magnon mode. We introduce the polarization $0 \le |\varepsilon_{\pm}| \le 1$ as the ellipticity of the mode, as will be specified below (see equation (9)). The maximal polarization corresponds to circularly polarized modes with an ellipticity $\varepsilon_{\pm} = 1$. The minimal polarization corresponds to linearly polarized modes with an ellipticity $\varepsilon_{\pm} = 0$. Calculations based on equation (6) show that the eigenmodes in presence of the field are circularly-polarized with field-dependent ellipticity

$$\varepsilon_{\pm} = \frac{4\omega_H \omega_{\pm}\left(\omega_{\pm}^2 - \omega_1^2\right)}{\left(\omega_{\pm}^2 - \omega_1^2\right)^2 + 4\omega_H^2 \omega_{\pm}^2}. \quad (9)$$

Each of the modes can carry field dependent angular momentum proportional to magnetization $m_{\pm} = \omega_{\pm}\varepsilon_{\pm} M_s / \gamma H_{ex}$. In contrast to the uniaxial case, ellipticities of the modes are different, $|\varepsilon_+| \ne |\varepsilon_-|$. The magnetizations of the modes differ not only in sign, but also in value, due to the difference of the frequencies. It should be noted that the absolute value of the angular

momentum is defined by the intensity of magnon fluctuations ($\propto \delta\mathbf{n}^2$) and depends on the external parameters (temperature or spin current).

Nonlocal transport. We associate R_{el} contribution with the current induced magnon spin current and R_{th} with spin Seebeck effect. The spin current in the Pt electrode modifies the effective damping coefficient of the spin-polarized modes and creates a nonequilibrium distribution of magnons with average magnetization $\mathbf{m}_{ave}$[2]

$$\mathbf{m}_{ave} = \frac{\gamma}{\alpha_G} M_s \, \mathbf{n}^{(0)} \left(\mathbf{H}_{curr} \cdot \mathbf{n}^{(0)}\right) \left[\varepsilon_+ \omega_+ f\left(\frac{\hbar\omega_+}{T}\right) + \varepsilon_- \omega_- f\left(\frac{\hbar\omega_-}{T}\right) \right], \quad (10)$$

where $f(\varepsilon) = \left[\exp\left(\varepsilon/\left(k_B T\right)\right) - 1\right]^{-1}$ is the Bose-Einstein equilibrium distribution function for each of the modes, $\hbar$ is the Planck constant, k_B is the Boltzmann constant. The gradient of the spin accumulation induces diffusion of spin polarized magnons to the detector electrode and is detected by the inverse spin-Hall effect. In this case $R_{\mathrm{el}} \propto \mathbf{m}_{ave} \cdot \mathbf{j}_{ISHE} \times \hat{z} \propto \left(\mathbf{n}^{(0)} \cdot \hat{\mu}\right)^2$, where $\mathbf{j}_{ISHE}$ is the current density in the detector electrode. As both electrodes for spin-pumping and measuring are parallel, $\mathbf{j}_{ISHE} \times \hat{z} \parallel \mathbf{H}_{curr} \parallel \hat{\mu}$.

Spin Seebeck effect is induced by the temperature gradient between the electrodes due to the Joule heating. In this case magnetization of the magnon is related with the field induced ellipticity of the magnon eigenmodes:

$$\mathbf{m}_{\mathrm{ave}} \propto \frac{\gamma}{\alpha_G} \mathbf{n}^{(0)} \left[\varepsilon_+ \omega_+ f\left(\frac{\hbar\omega_+}{T}\right) - \varepsilon_- \omega_- f\left(\frac{\hbar\omega_-}{T}\right) \right]. \quad (11)$$

Correspondingly, $R_{\mathrm{th}} \propto \mathbf{m}_{ave} \cdot \mathbf{j}_{ISHE} \times \hat{z} \propto \left(\mathbf{n}^{(0)} \cdot \hat{\mu}\right)$.

References

1 Coppens, P. & Eibschütz, M. Determination of the crystal structure of yttrium orthoferrite and refinement of gadolinium orthoferrite. *Acta Crystallogr.* **19**, 524-531 (1965).

2 Lebrun, R. *et al.* Long-distance spin-transport across the Morin phase transition up to room temperature in ultra-low damping single crystals of the antiferromagnet α-Fe_2O_3. *Nat. Commun.* **11**, 1-7 (2020).

3 Uchida, K. *et al.* Spin Seebeck insulator. *Nat. Mater.* **9**, 894-897 (2010).

4 Xiao, J., Bauer, G. E. W., Uchida, K.-c., Saitoh, E. & Maekawa, S. Theory of magnon-driven spin Seebeck effect. *Phys. Rev. B* **81**, 214418 (2010).

5 Cornelissen, L. J., Liu, J., Duine, R. A., Youssef, J. B. & Van Wees, B. J. Long-distance transport of magnon spin information in a magnetic insulator at room temperature. *Nat. Phys.* **11**, 1022-1026 (2015).

6 Lebrun, R. *et al.* Tunable long-distance spin transport in a crystalline antiferromagnetic iron oxide. *Nature* **561**, 222-225 (2018).

7 Sinova, J., Valenzuela, S. O., Wunderlich, J., Back, C. & Jungwirth, T. Spin hall effects. *Rev. Mod. Phys.* **87**, 1213 (2015).

8 Rezende, S. M., Azevedo, A. & Rodriguez-Suarez, R. L. Magnon diffusion theory for the spin Seebeck effect in ferromagnetic and antiferromagnetic insulators. *J. Phys. D: Appl. Phys.* **51**, 174004 (2018).

9 Li, J. *et al.* Spin current from sub-terahertz-generated antiferromagnetic magnons. *Nature* **578**, 70-74 (2020).

10 Reitz, D., Li, J., Yuan, W., Shi, J. & Tserkovnyak, Y. Spin Seebeck effect near the antiferromagnetic spin-flop transition. *Phys. Rev. B* **102**, 020408 (2020).

11 Jansen, R. *et al.* Superimposed contributions to two-terminal and nonlocal spin signals in lateral spin-transport devices. *Phys. Rev. B* **104**, 144419 (2021).

12 Jacobs, I. S., Burne, H. F. & Levinson, L. M. Field-induced spin reorientation in $YFeO_3$ and $YCrO_3$. *J. Appl. Phys.* **42**, 1631-1632 (1971).

13 Judin, V. M., Sherman, A. B. & Myl'Nikova, I. E. Magnetic properties of $YFeO_3$. *Phys. Lett.* **22**, 554-555 (1966).

14 Lin, X., Jiang, J., Jin, Z. & Ma, G. in *2015 40th International Conference on Infrared, Millimeter, and Terahertz waves (IRMMW-THz).* 1-1 (IEEE).

15 Jin, Z. *et al.* Single-pulse terahertz coherent control of spin resonance in the canted antiferromagnet $YFeO_3$, mediated by dielectric anisotropy. *Phys. Rev. B* **87**, 094422 (2013).

16 Gomonay, O., Yamamoto, K. & Sinova, J. Spin caloric effects in antiferromagnets assisted by an external spin current. *J. Phys. D: Appl. Phys.* **51**, 264004 (2018).

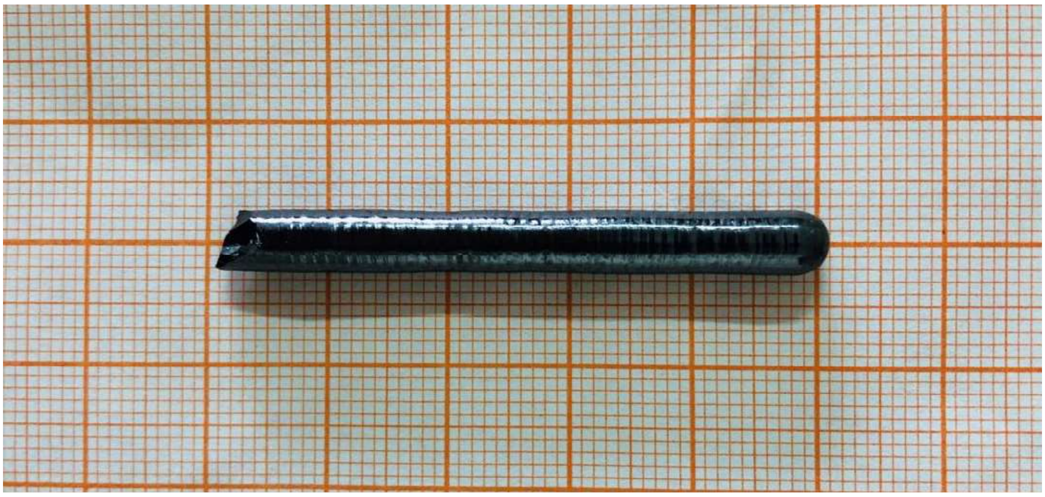

Supplementary Figure 1: Single Crystal. Picture of $YFeO_3$ single crystal grown by optical float-zone method.

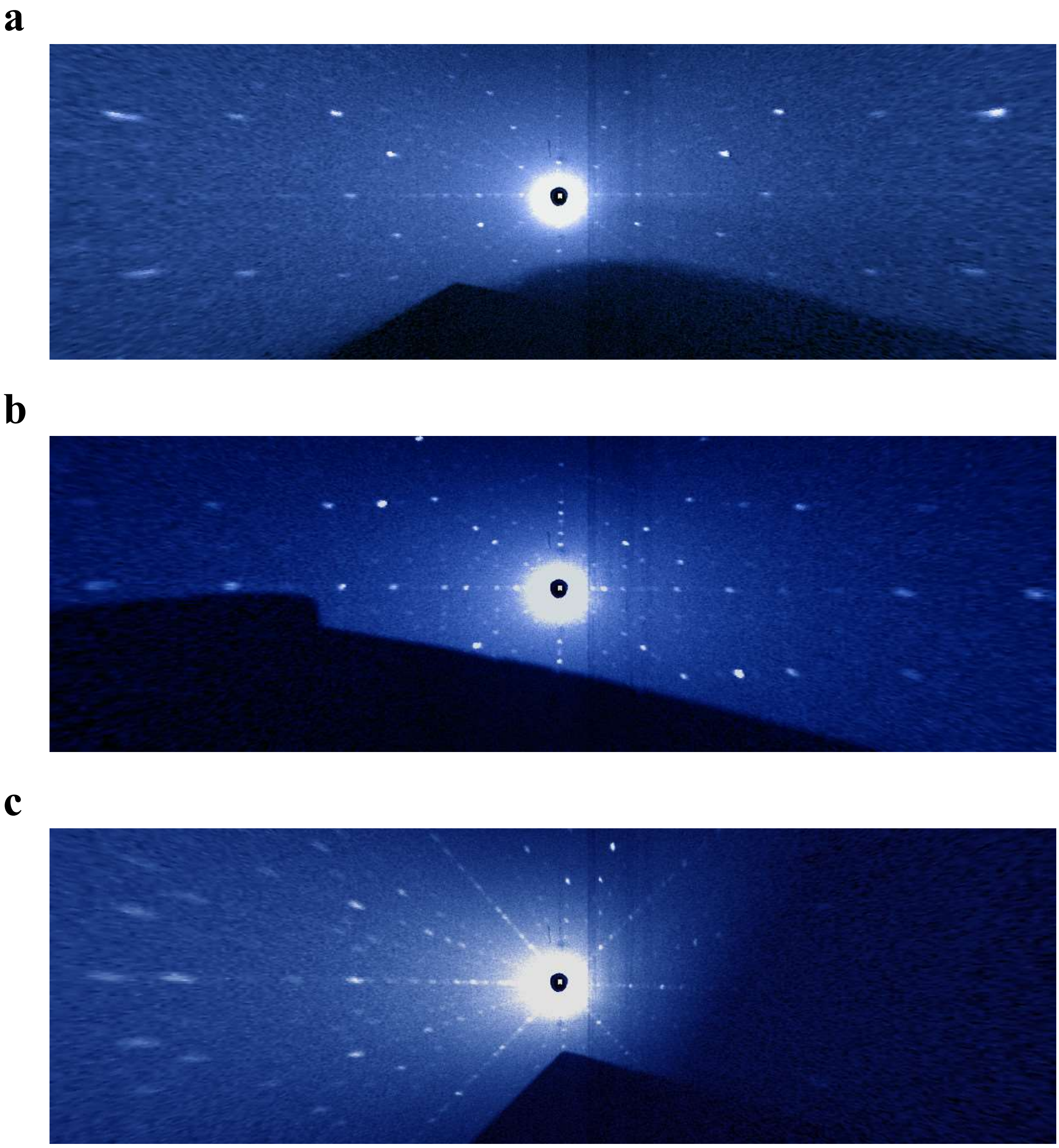

Supplementary Figure 2: Laue X-ray photography. Laue photography of $YFeO_3$ single crystal along the (**a**) *a*-, (**b**) *b*-, and (**c**) *c*- axis.

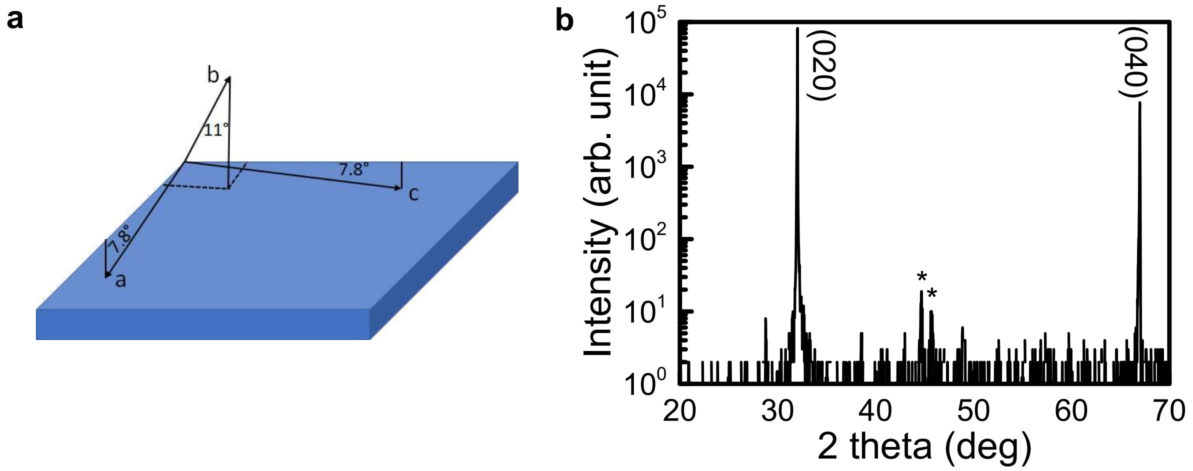

Supplementary Figure 3: Sample orientation. (**a**) Schematics of crystallographic axes with respect to sample plane due to miscut. (**b**) θ-2θ scan measured along (010) direction perpendicular to the surface of the sample plane. Only (020) and (040) peaks are observed and levelled accordingly. Peaks marked with a star come from aluminium sample holder and are not related to the $YFeO_3$ crystal.

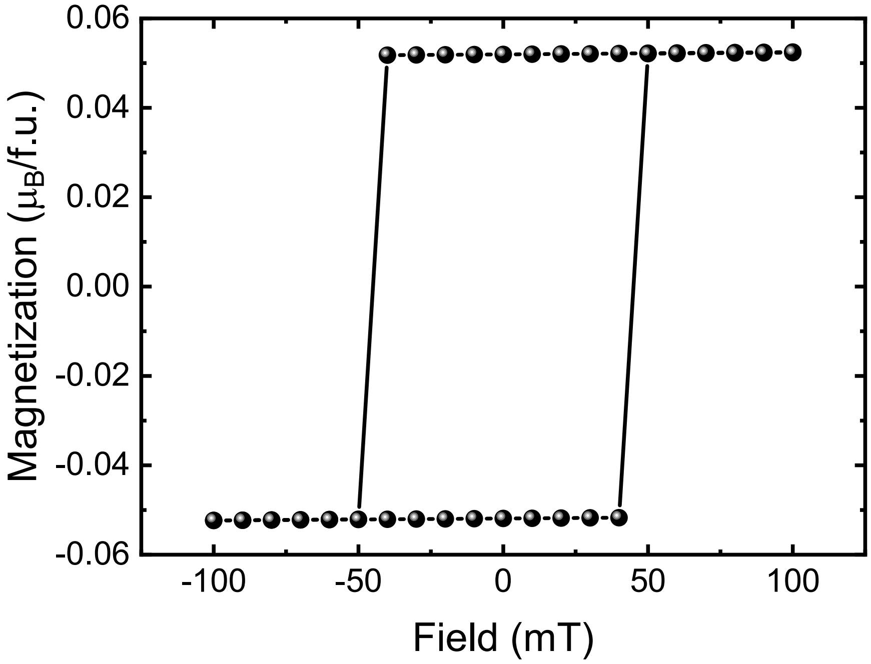

Supplementary Figure 4: Magnetization curve. The *m-H* hysteresis loop for field applied along *c*-axis.

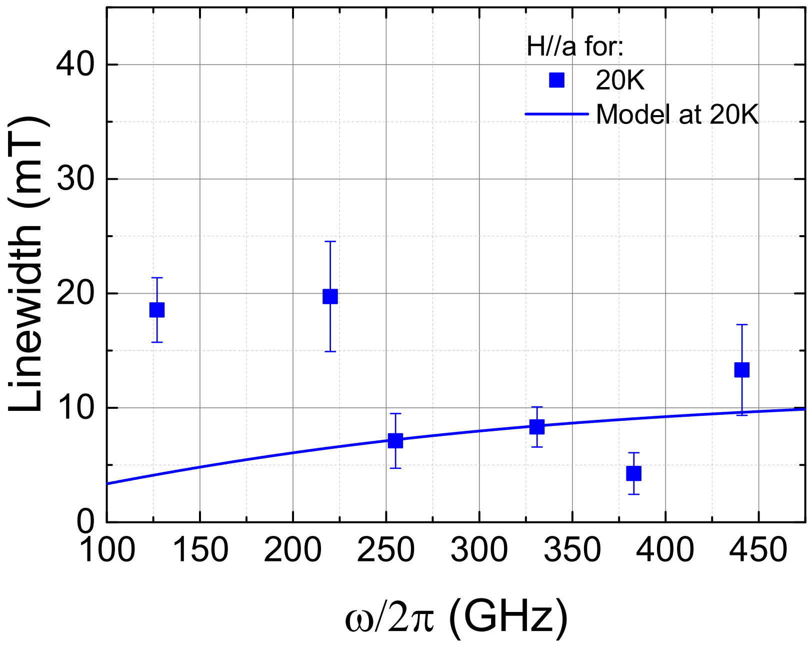

Supplementary Figure 5: Damping coefficient. Linewidth as a function of frequency for the configuration of H along the a-axis (easy axis) above the spin flop field. The blue points correspond to experimental measurements at 20K. The model gives a Gilbert damping coefficient of $6 \pm 2 \times 10^{-6}$ for 20K. The extracted parameters from Figure 1(b) of main text, $\mu_0 H_E = 635\ T$, $\mu_0 H_a = 0.19\ T$, $\mu_0 H_b = 0.7\ T$ and $\mu_0 H_{DMI} = 12\ T$, are used in this model.

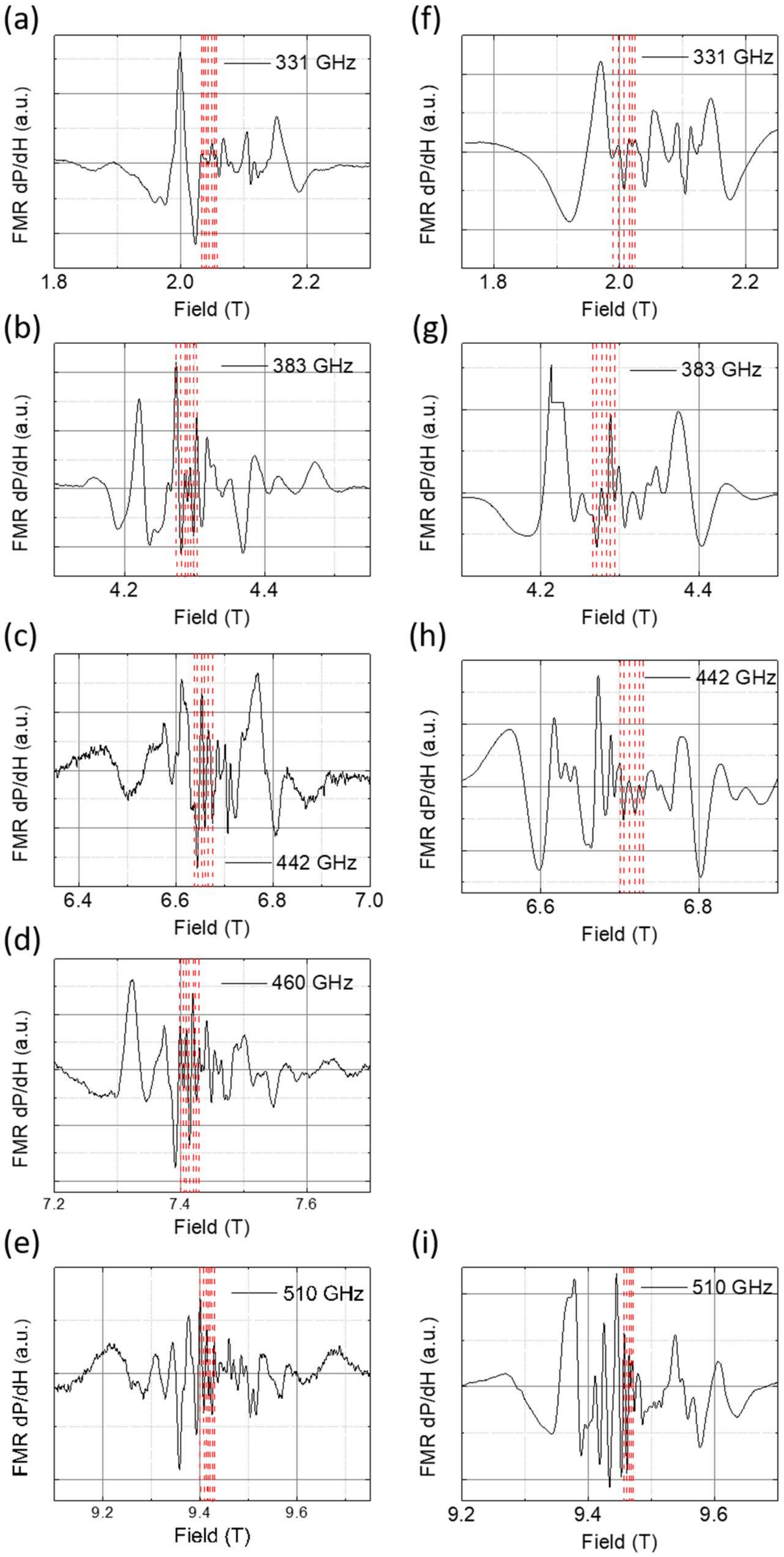

Supplementary Figure 6: AFMR resonance for the magnetic field applied along the c-axis at 20 K. Resonance peaks at different frequencies for a 0.5 mm thick $YFeO_3$ (0.5 mm)/ Pt (5 nm) sample for the configuration of H along the c-axis (intermediate axis) at 20K, for two sets of data (columns a-e) and (columns f-i) taken separately after removal and reintroduction of the sample. The resonance FWHM linewidth is extracted by measuring the average peak-to-peak distance of the resonances, indicated by the red dashed lines.

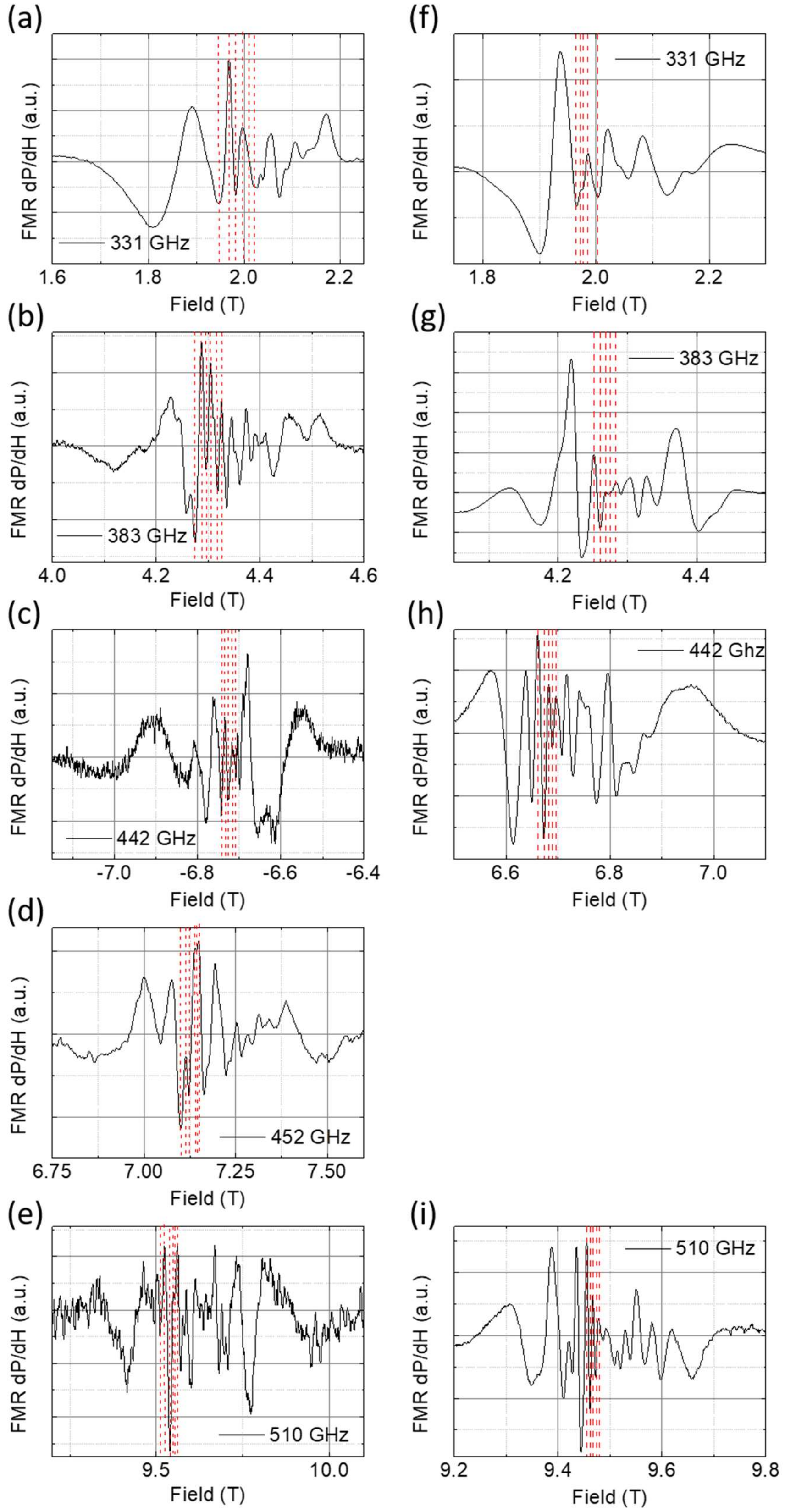

Supplementary Figure 7: AFMR resonance for the magnetic field applied along the c-axis at 150 K. Resonance peaks at different frequencies for a 0.5 mm thick $YFeO_3$ (0.5 mm) / Pt (5 nm) sample for the configuration of H along the c-axis (intermediate axis) at 150K, for two sets of data (columns a-e) and (columns f-i) taken separately after removal and reintroduction of the sample. The resonance FWHM linewidth is extracted by measuring the average peak-to-peak distance of the resonances, indicated by the red dashed lines.

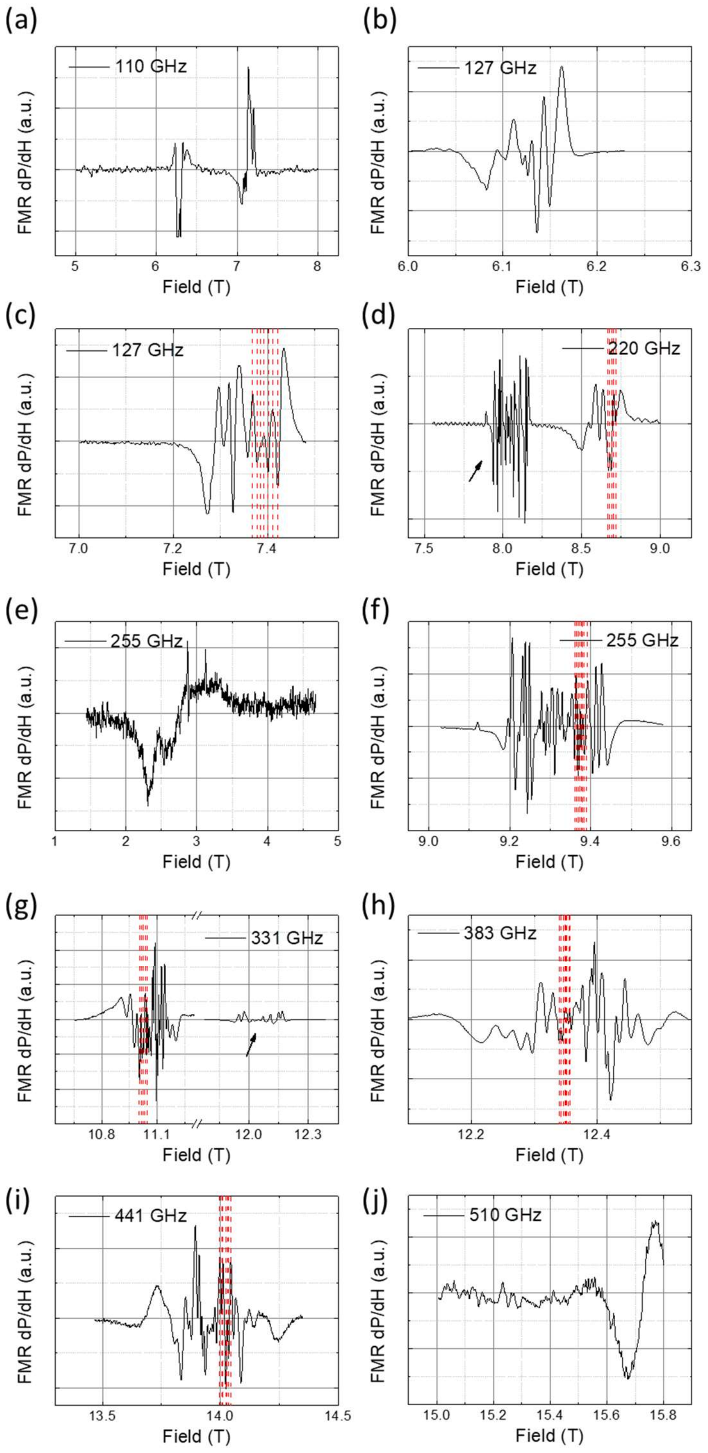

Supplementary Figure 8: AFMR resonance for the magnetic field applied along the a-axis at 20 K. Resonance peaks at different frequencies for a 0.5 mm thick $YFeO_3$ / Pt (5 nm) sample. (a) 110 GHz, (b) 127 GHz, (c) 220 GHz, (d) 255 GHz, (e) 331 GHz with two sets of data that shows additional resonances at higher field related to g=2, (f) 383 GHz, (g) 441 GHz, (h) 510 GHz. The resonance FWHM linewidth is extracted by measuring the average peak-to-peak distance of the resonances above the critical field value, indicated by the red dashed lines. Note that there are two resonances at (d) and (g); A control experiment was made with just the sample holder that unraveled an additional signal (marked with an arrow) not related to the bench, near the paramagnetic resonance g=2 (see also the black dashed line in Figure 1(b) of main text). For the extraction of the damping, only the other resonance peaks are used that fall on the fitting curve.

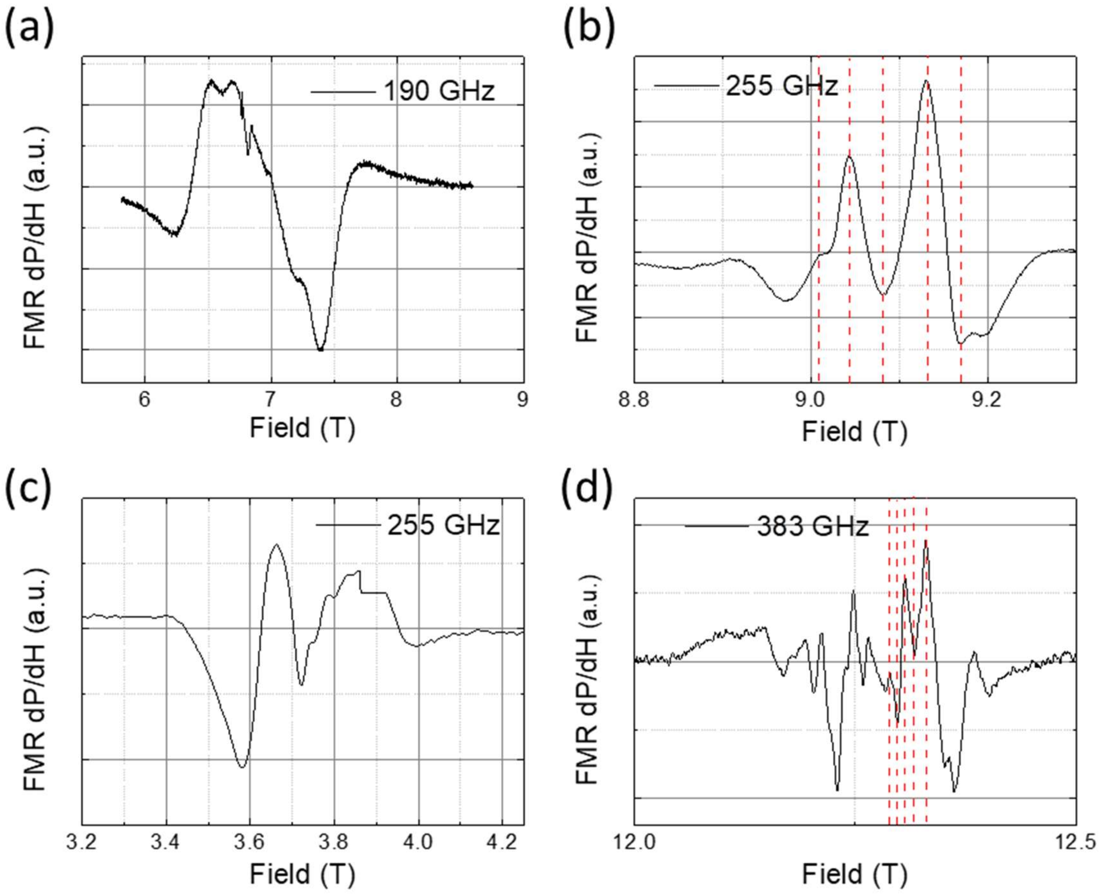

Supplementary Figure 9: AFMR resonance for the magnetic field applied along the a-axis at 150 K. Resonance peaks at different frequencies for a 0.5 mm thick $YFeO_3$ / Pt (5 nm) sample. (a) 190 GHz, (b) 255 GHz, (c) 255 GHz, (d) 383 GHz. The resonance FWHM linewidth is extracted by measuring the average peak-to-peak distance of the resonances above the critical field value, indicated by the red dashed lines.

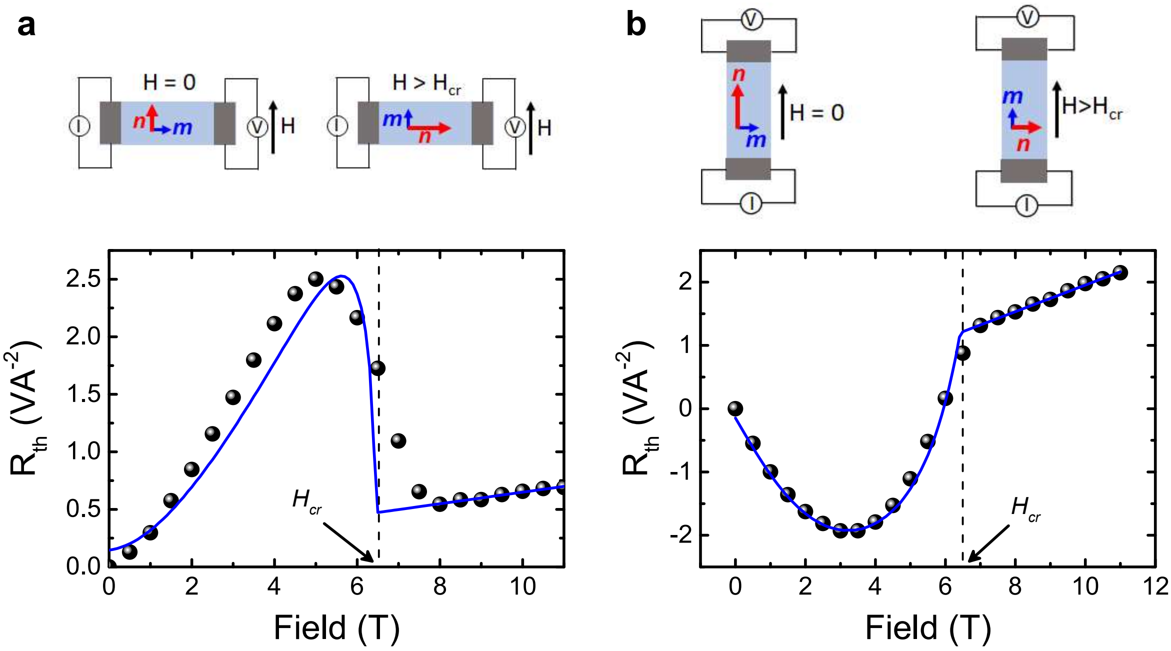

Supplementary Figure 10: Thermal spin transport at 200 K. (**a**) R_{th} as function of field applied along the easy-axis in device where wires are parallel to the easy-axis. R_{th} increases with field and reaches maximum below H_{cr} followed by a sharp decrease and above H_{cr} a linear field dependence is observed. (**b**) R_{th} as function of field applied along the easy-axis in device where wires are perpendicular to the easy-axis. R_{th} increase with field to reach a maximum at 3 T followed by a change of sign below H_{cr} and finally a change in gradient above H_{cr}. In both curves, the signal is plotted after subtracting the offset at zero field value. The solid lines correspond to the theoretical model are described in the text. The error bars are smaller than the symbols.

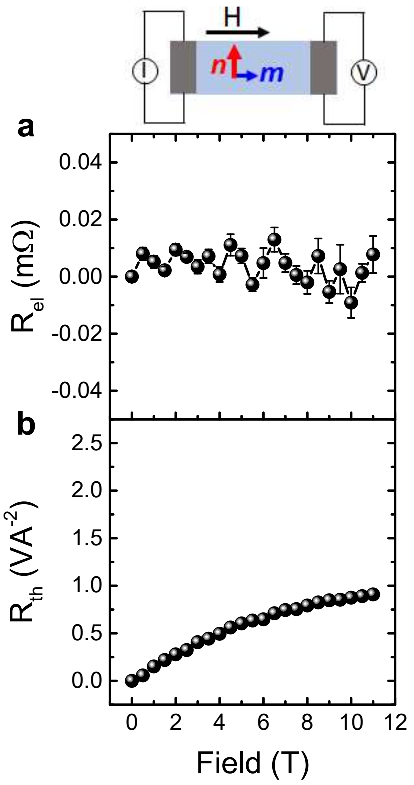

Supplementary Figure 11: Spin transport for wires along the easy-axis and field perpendicular to the easy-axis. (**a**) R_{el} signal is plotted as a function of field perpendicular to the easy-axis. No spin transport signal is observed in the whole field range. The error bars are calculated from the standard deviation of the mean. (**b**) R_{th} as a function of field shows a significant increase with field and a saturation tendency at higher field. The error bars are within the symbol height. The schematics is showing the direction of Néel vector, magnetization and field direction with respect to the wire.

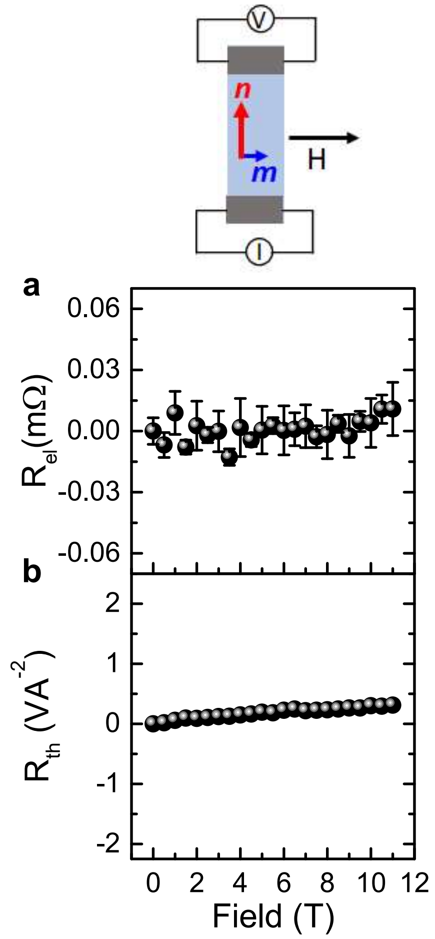

Supplementary Figure 12: Spin transport for wires perpendicular to the easy-axis and field perpendicular to the easy-axis. Schematics shows the relative orientation of the wire, Néel vector, magnetization and applied magnetic field. (**a**) R_{el} vs H, where no spin signal is observed in whole field range from 0 to 11 T. (b) Weak increase in R_{th} as a function of field.

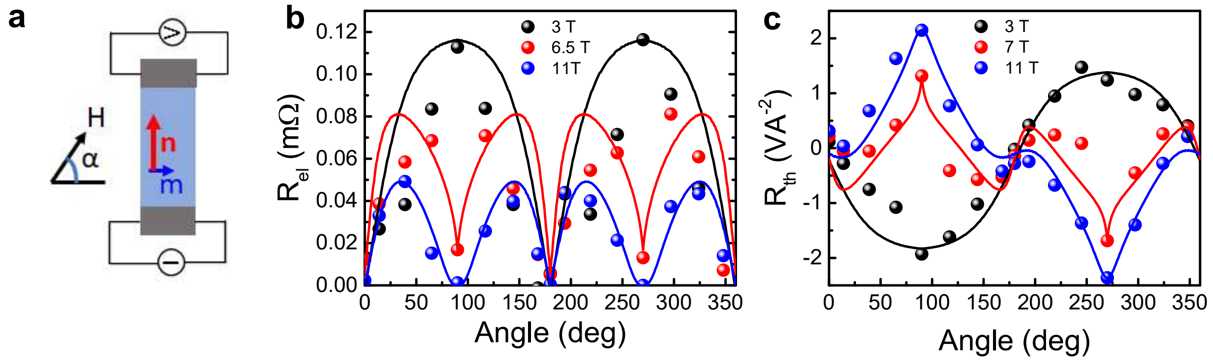

Supplementary Figure 13: Angular dependence of spin transport signal. (**a**) Schematics of the device and the measurement geometry along with the direction of Néel vector and weak magnetization at zero magnetic field are shown. (**b**) and (**c**) Angular dependence of R_{el} and R_{th} signal, respectively, for various magnetic fields. The solid lines are the fitting based on magnon dynamics from the proposed theoretical model. A constant offset is subtracted from the experimental data.

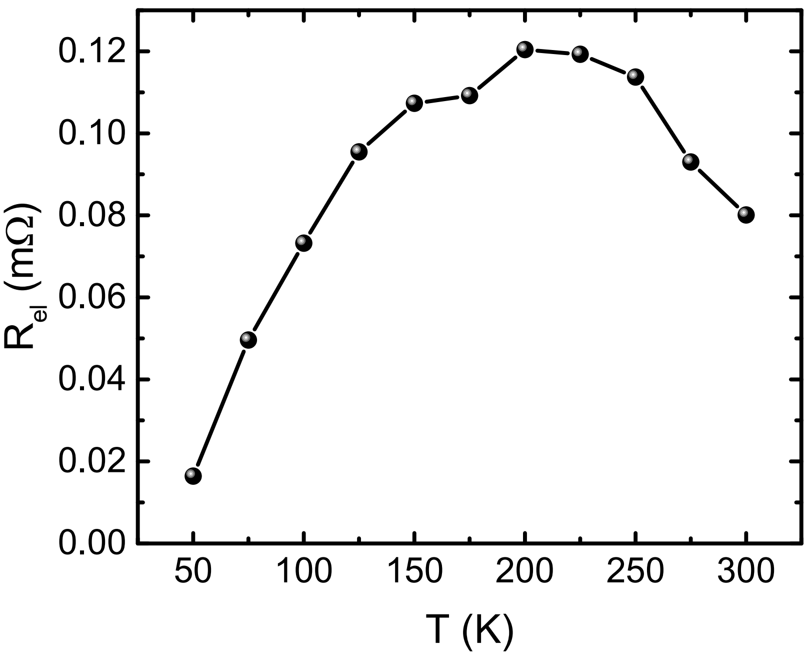

Supplementary Figure 14: Temperature dependence of spin transport. Spin transport signal as a function of temperature at $H = 3$ T for device where wires are perpendicular to the easy-axis.
